# Supplementary material for: PMEL is involved in snake colour pattern transition from blotches to stripes
Source: Nat Commun. 2024 Sep 3;15:7655. doi: 10.1038/s41467-024-51927-0 (PMC11371805; doi:10.1038/s41467-024-51927-0)
Supplement: Supplementary file 1 — Supplementary Information [file 41467_2024_51927_MOESM1_ESM.pdf]

Supplementary Information for

PMEL is involved in snake color pattern transition from blotches to stripes

Athanasia C. Tzika[1*], Asier Ullate-Agote[1,2], Pierre-Yves Helleboid[1] and Maya Kummrow[3]

[1] Laboratory of Artificial & Natural Evolution (LANE), Department of Genetics & Evolution, University of Geneva, Geneva, Switzerland

[2] current address: Biomedical Engineering Program, Center for Applied Medical Research (CIMA), Universidad de Navarra, Instituto de Investigación Sanitaria de Navarra (IdiSNA), Pamplona, Spain

[3] Tierspital, University of Zurich, Zurich, Switzerland

***Correspondance:**

athanasia.tzika@unige.ch

Supplementary text
Staging of the corn snake embryos
The embryos used for transcriptomic analyses and whole-mount in situ hybridisations were staged as described here and roughly based on the staging of *Boaedon* (*Lamprophis*) *fuliginosus*[1].

Stage S2
The body forms four to five tight coils. The eye is lightly pigmented. The remnants of two pharyngeal slits are visible and the frontonasal prominence has a squared shape. There are still no visible endolymphatic ducts. The tip of the lower jaw is anterior to the eye.
Stage S3
The body forms four to five tight coils up to the cloaca. Bulges are observed ventrally, where the ventral scales will form, and the coelum is open along the entire body. The heart and liver are external. The endolymphatic duct spots are visible as white dots, and the diencephalon is smaller than the mesencephalon. The lower jaw is smaller than 1/3 of the upper jaw. The eye is pigmented with the choroid fissure evident. One pharyngeal slit is visible.
Stage S4
The diencephalon has a similar size as the mesencephalon. The lower jaw has similar length as the upper jaw. The choroid fissure is not visible.
Stage S5
The eye lid/scale starts to close and the skin and bone over the brain are more opaque. The lateral placodes start to form.
Stage S6
The body forms 3.5 coils and the coelum is almost closed from the head to the heart region. The heart and liver are partially internalised. The lateral placodes are fully formed.
Stage S7
The body forms 3 coils and the coelum is closed from the head to the heart regions. The heart and liver are internalised but still visible. The dorsal placodes start to form.
Stage S8
The body is fully scaled laterally. The coelum is closed along two thirds of the body length. The heart and liver are no longer visible. The ventral scales near the head are fused. The mouth starts to open, and the tongue is visible.
Stage S9
Dorsal scales start to form near the head, and the ventral scales are fused along two thirds of the body length. The ventral scales start to become asymmetrical. The coelum is open near the cloaca and the intestine is visible.
Stage S10
The dorsal scales are formed along two thirds of the body length. All ventral scales are fused.

Reference
1 Boback, S. M., Dichter, E. K. & Mistry, H. L. A developmental staging series for the African house snake, Boaedon (Lamprophis) fuliginosus. *Zoology* **115**, 38-46, doi:10.1016/j.zool.2011.09.001 (2012).

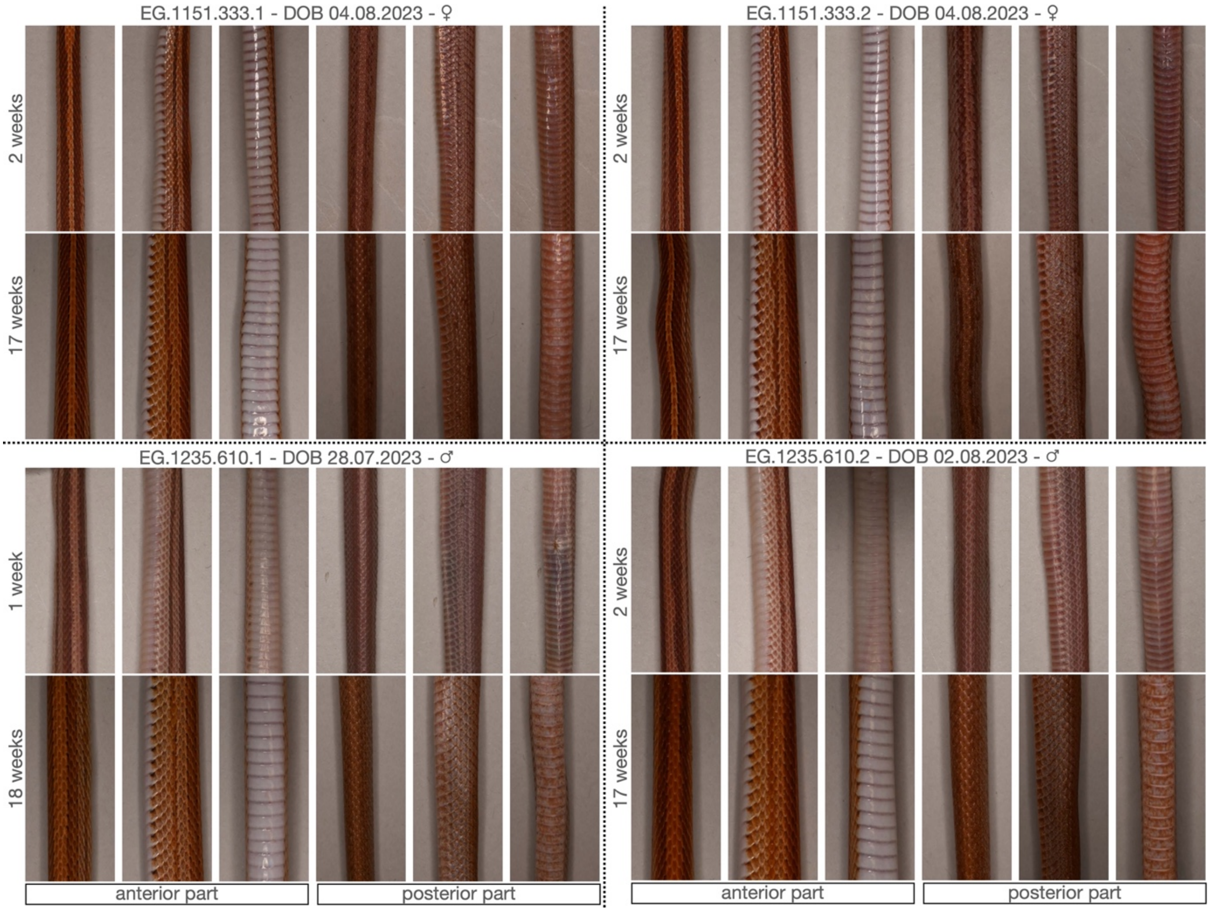

Supplementary Figure 1. Imaging of four Terrazzo individuals. Photos of the dorsal, lateral, and ventral side from the anterior part of the body (near the head) and the posterior part of the body (near the cloaca) of four Terrazzo individuals at two different time points post hatching.

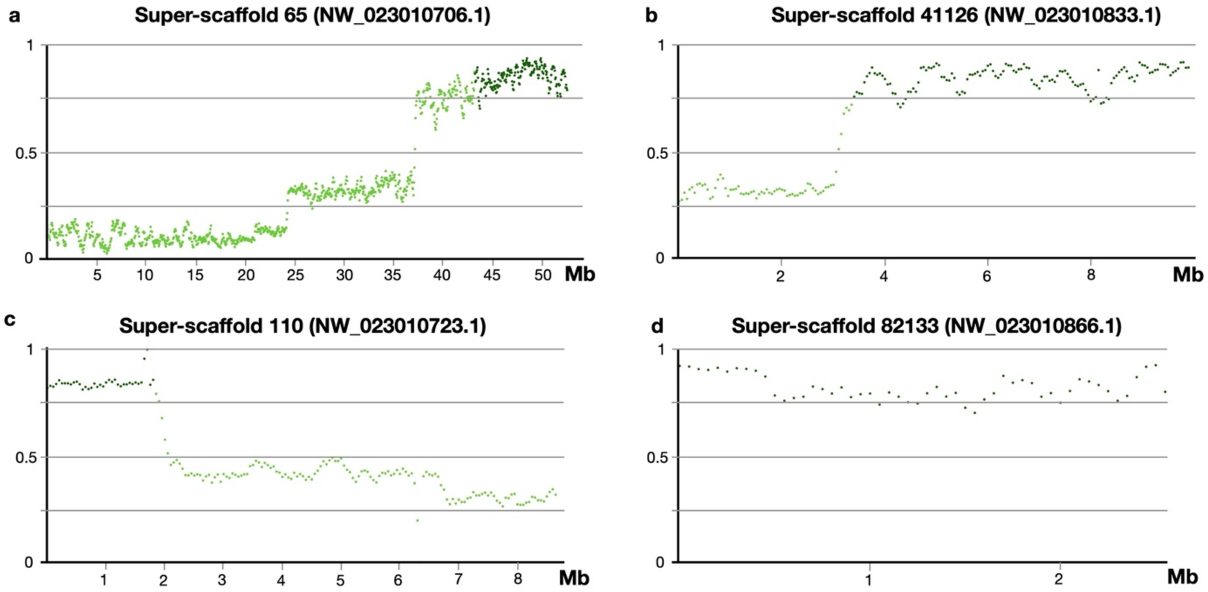

Supplementary Figure 2. Additional scaffolds presenting a high proportion of cosegregating variants. Proportion of biallelic variants (SNP/MNP and indels) cosegregating with the Terrazzo locus considering a 200-kb sliding window and a step of 50 kb in Super-scaffold 65 (a), Super-scaffold 41126 (b), Super-scaffold 110 (c) and Super-scaffold 82133 (d). In none of these scaffolds do we observe regions fully cosegregating with the Terrazzo locus, as is the case for Super-scaffold 85.

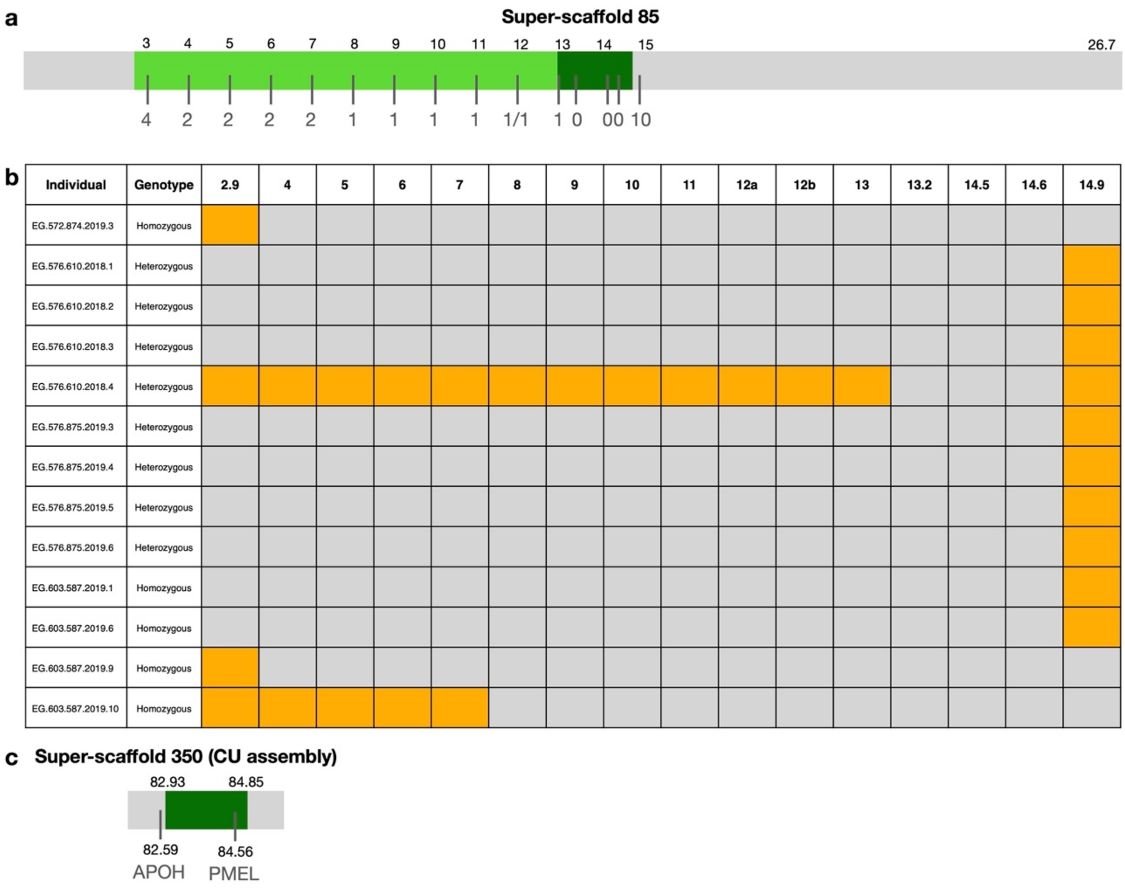

Supplementary Figure 3. Reduction of the genomic interval harbouring the Terrazzo variant. (a) Schematic representation of Super-scaffold 85 and the positions (in Mb) where recombination events were detected. The numbers below the scaffold detail the number of recombinants at each position. In dark green, we highlight the reduced interval. (b) List of the 13 recombinant individuals (among 91) and the genotyping results. Recombined sites are in orange. (c) Position of the reduced interval on the CU assembly.

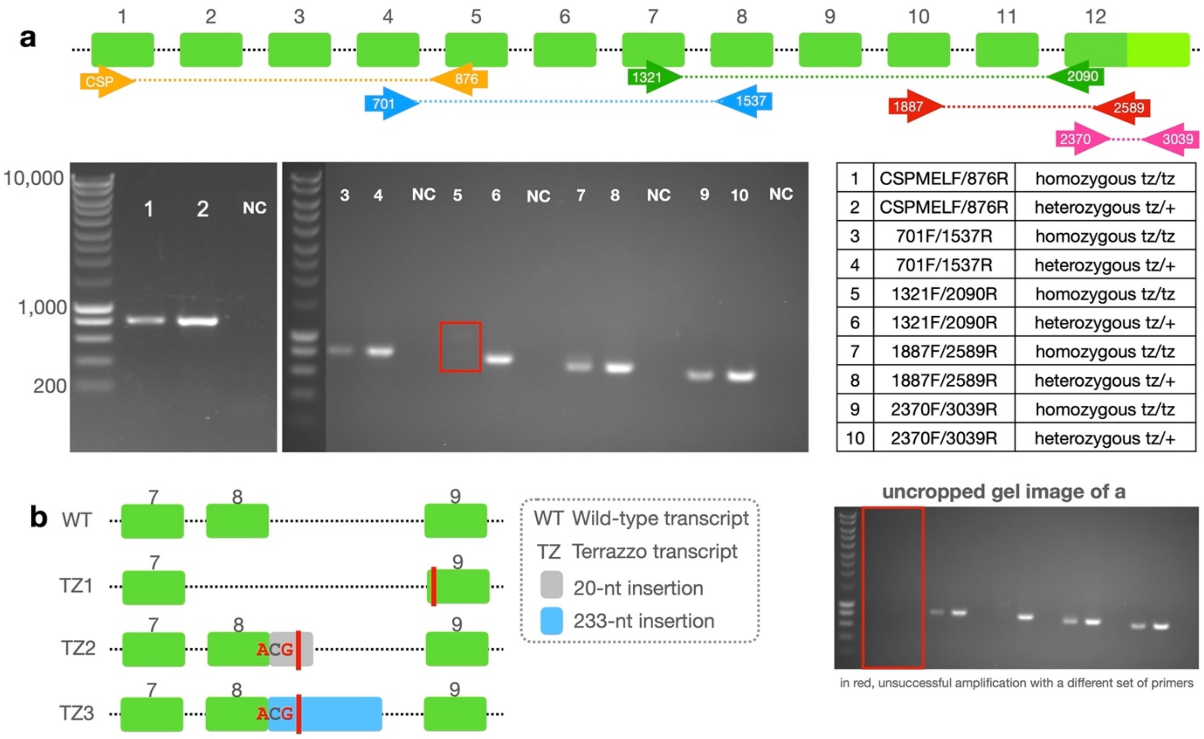

Supplementary Figure 4. Amplification of the *PMEL* transcript. (a) Amplification of the *PMEL* transcript from a homozygous and a heterozygous individual with five overlapping fragments. The DNA ladder ranges from 200 to 10,000 bp (SmartLadder SF, Eurogentec). NC: PCR negative control (b) Schematic representation of the WT *PMEL* transcript and the three *PMEL* isoforms detected in Terrazzo individuals (TZ1, TZ2, and TZ3). The red lines indicate premature STOP codons. We cannot exclude the presence of additional isoforms.

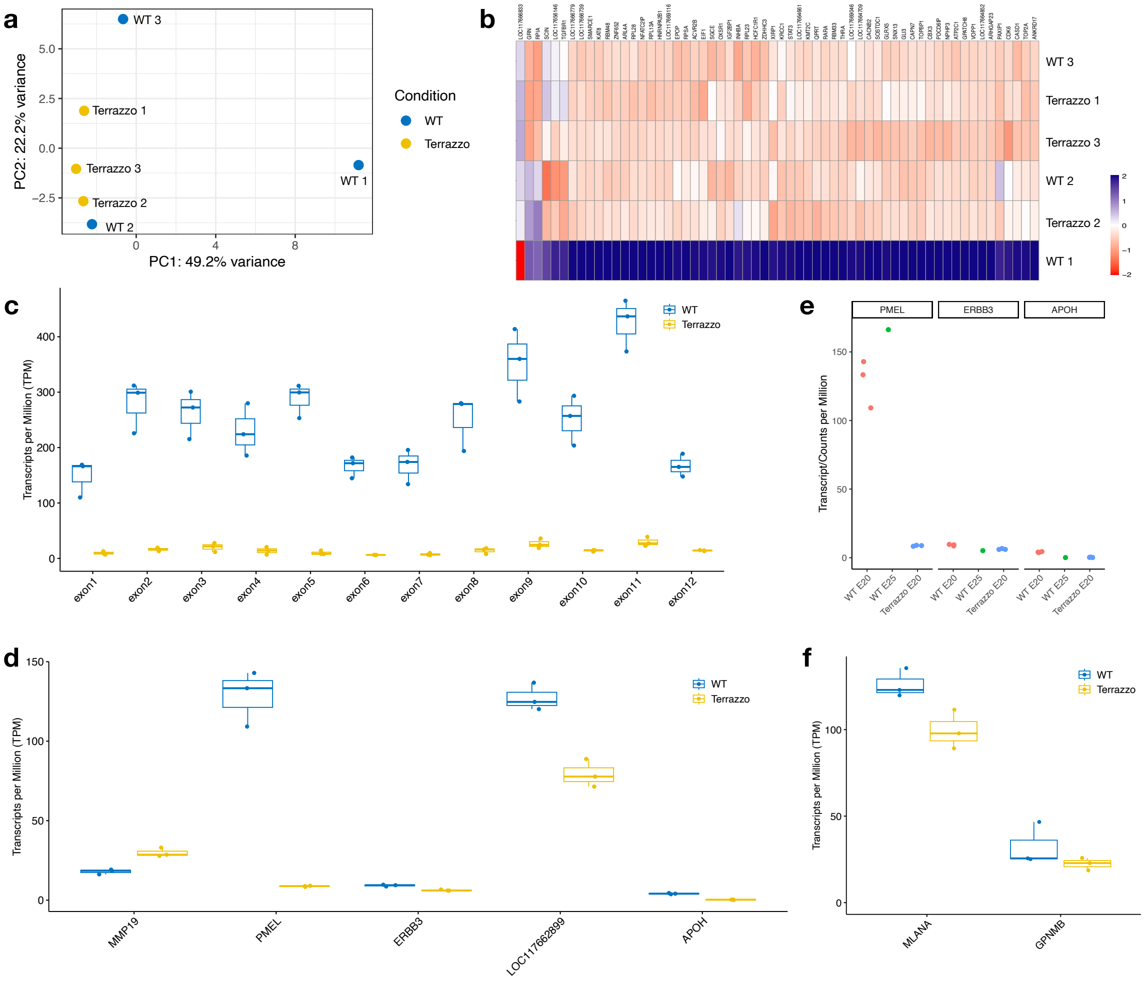

Supplementary Figure 5. RNA-seq gene expression analyses. (a) Principal component analyses of the three wild-type and the three Terrazzo samples used for RNA-seq. (b) Heatmap of the 60 most differentially expressed genes located on the Z chromosome (based on *Crotalus viridis* Z chromosome; NCBI accession CM012323.1). Given that corn snake males are homogametic (ZZ), it is clear from this comparison that sample WT1 is the only male sampled. This is the main biological difference responsible for the clustering of the samples in the PCA presented in (a). We do not expect clustering based on the coloration at this stage, because the chromatophores are at a very early stage of differentiation and no coloration is visible. (c) Transcripts per million (TPM) calculations for the 12 exons of *PMEL*, corrected for exon and library size. (d) TPMs for the genes in the Terrazzo interval with an adjusted p-value <0.05. We include the *APOH* gene which is located near the interval, and it is significantly downregulated in Terrazzo. The gene *LOC117662899* has been replaced by *LOC117662900* in NCBI and it corresponds to an uncharacterized non-coding RNA. (e) Comparison of the TPMs for *PMEL, ERBB3*, and *APOH* as calculated from the bulk RNAseq at E20 (S7) and the CPMs as calculated from single-cell dataset sampled at E25 (S9). (f) TPMs from the bulk RNAseq for *MLANA* and *GPNMB*, both used to perform whole-mount in situ hybridization experiments. The elements of the box plots are: centre line, median; box limits, upper and lower quartiles; whiskers, 1.5x interquartile range; points, measurements). Source data are provided as a Source Data file.

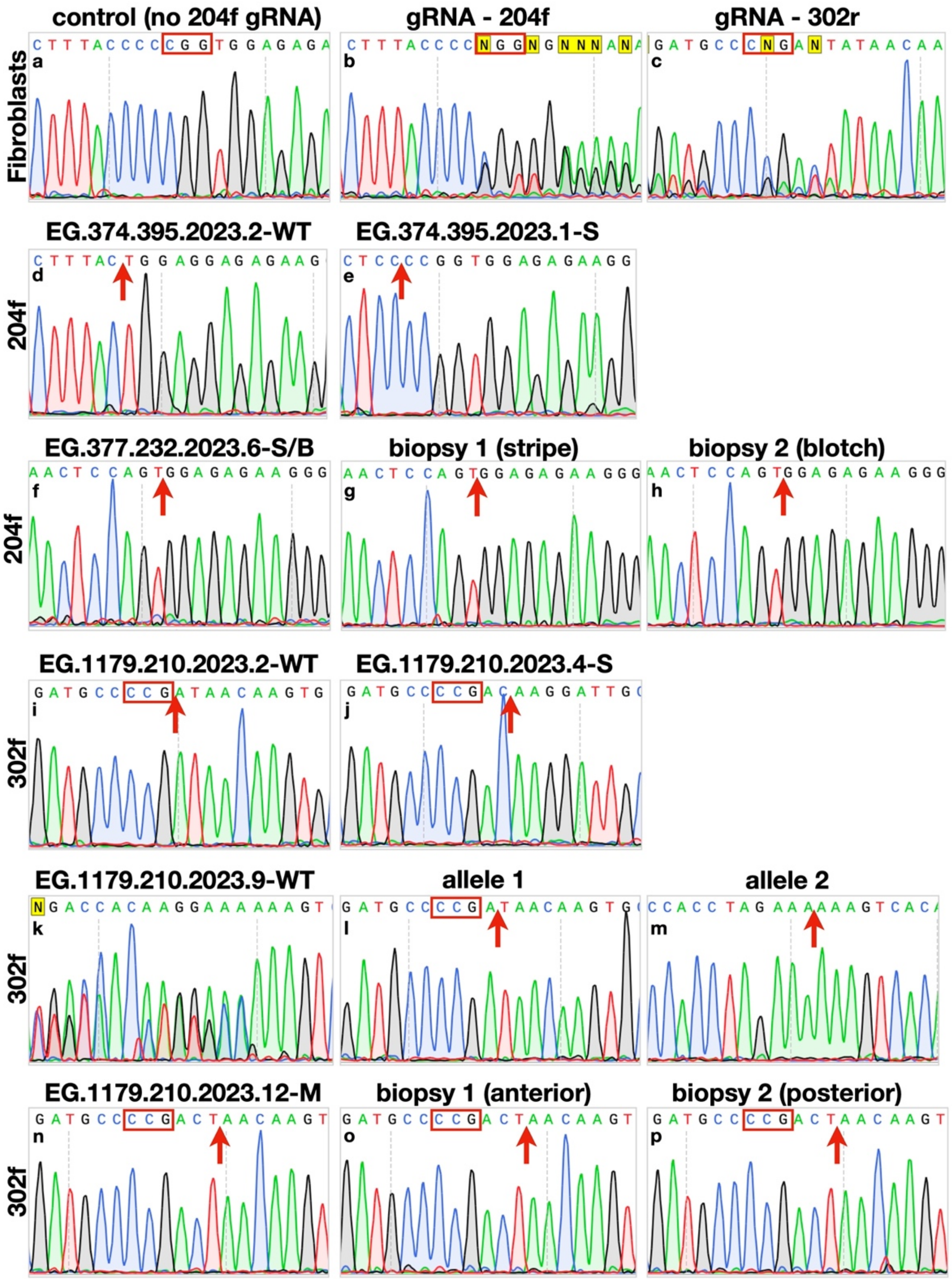

Supplementary Figure 6. Sequencing results of the *PMEL* target regions for functional analyses. Electropherogram of the target region sequenced on DNA extracted from (a) control corn snake fibroblasts that were transfected with a blank (no gRNA), (b) corn snake fibroblasts transfected with gRNA 204f, (c) corn snake fibroblasts transfected with gRNA 302r, (d) corn snake *PMEL* mutant with a wild type phenotype (204f), (e) corn snake *PMEL* mutant with stripes (204f), (f) corn snake *PMEL* mutant with both stripes and blotches (204f), (g) biopsy 1 taken from (f) from a region with stripes, (h) biopsy 2 taken from (f) from a region with blotches, (i) corn snake *PMEL* mutant with a wild type phenotype (302r), (j) corn snake *PMEL* mutant with stripes (302r), (k) corn snake *PMEL* mutant with a wild type phenotype (302r) and heterozygous, (l) cloned allele 1 from (k), (m) cloned allele 2 from (k), (n) corn snake *PMEL* mutant with a modified phenotype (302r), (o) biopsy 1 taken from (n) from an anterior region, (p) biopsy 2 taken from (n) from a posterior region. WT: wild type, S: stripes, B: blotches, red rectangles: PAM site, red arrows: insertion/deletion sites.

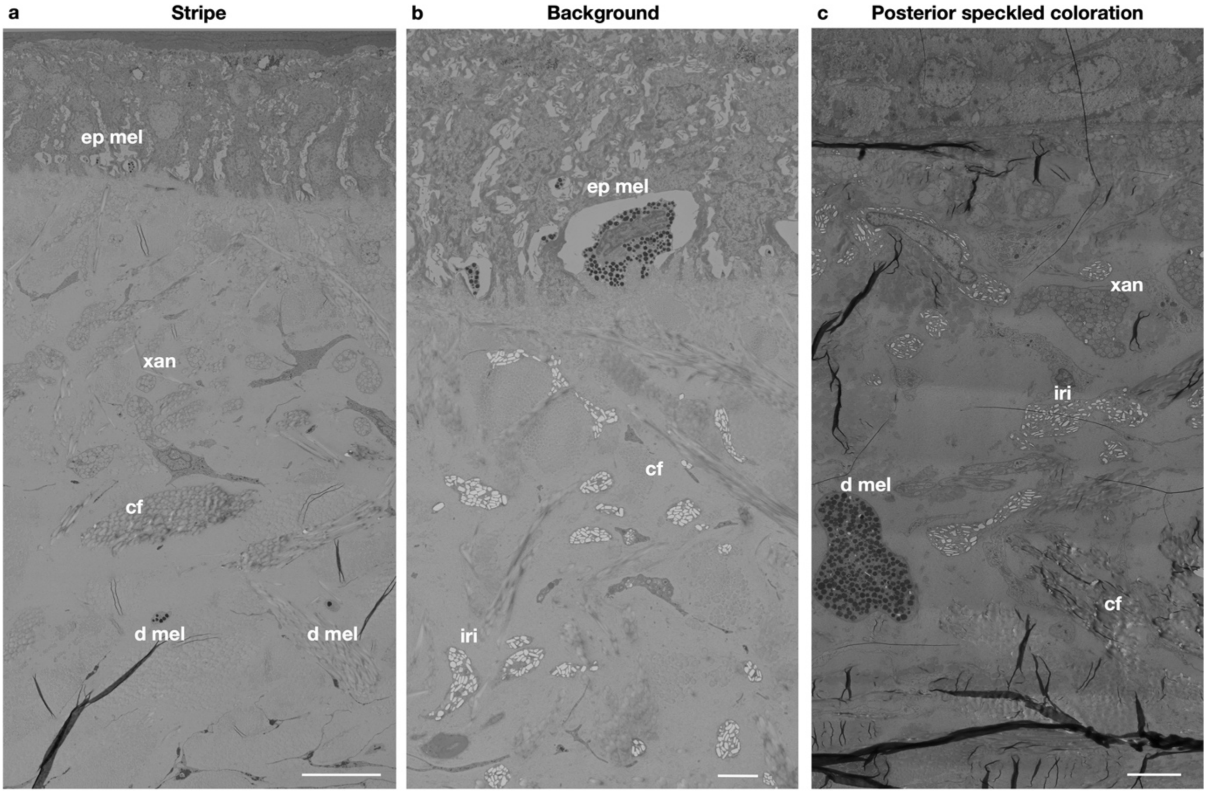

Supplementary Figure 7. TEM imaging of Terrazzo adult skin. Micrograph of (a) the red dorsal stripe, (b) the background region (between the stripes), and (c) the posterior body skin with speckled coloration. Three blocks of each type of sample were prepared. ep mel: epidermal melanophore, d mel: dermal melanophore, xan: xanthophore, iri: iridophore, cf: collagen fibers. Scale bar: 5 µm.

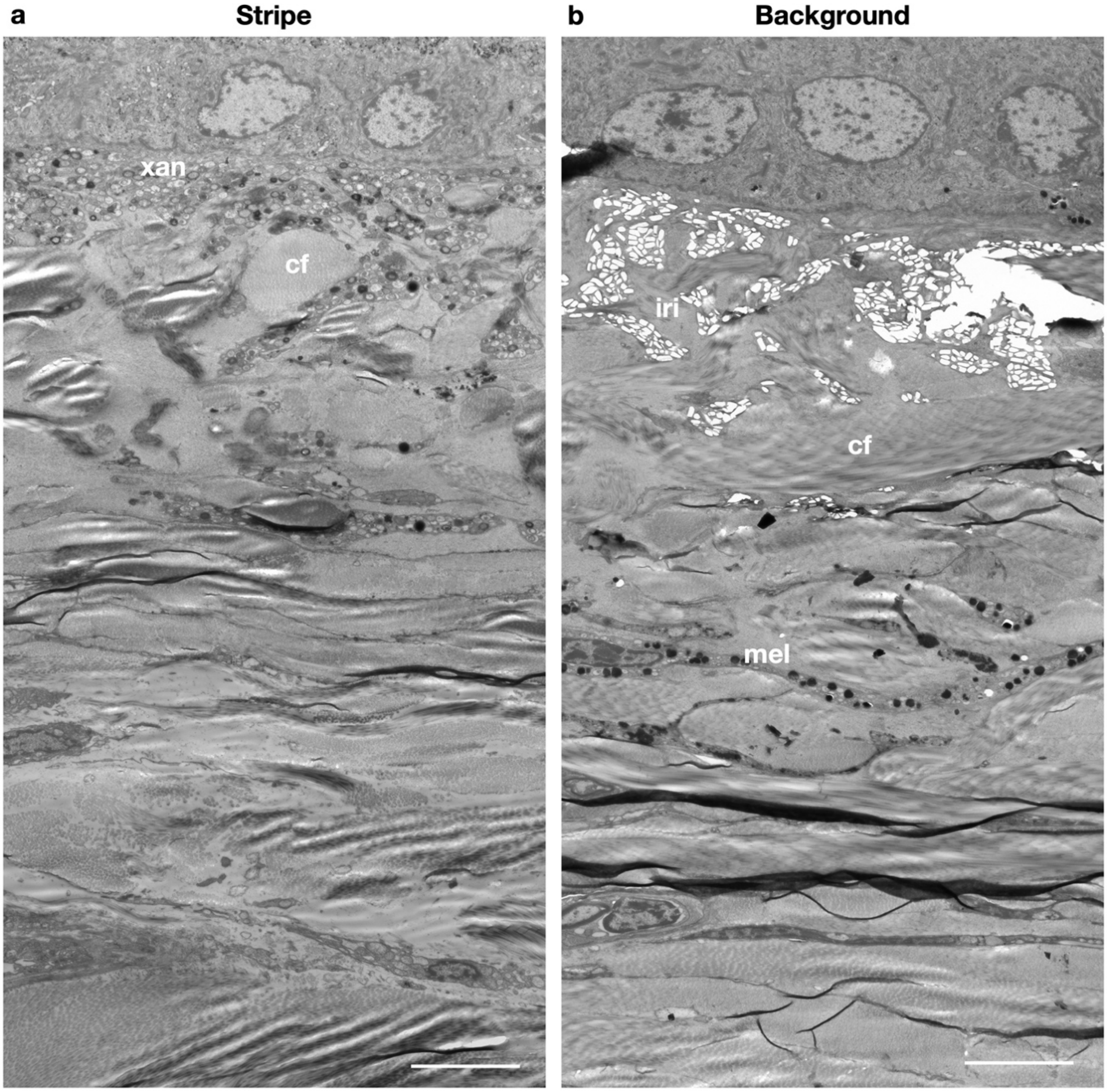

Supplementary Figure 8. TEM imaging of the *PMEL* mutant skin. Micrograph of (a) the red dorsal stripe, and (b) the background region (between the stripes). Three blocks of each type of sample were prepared. mel: melanophore, xan: xanthophore, iri: iridophore, cf: collagen fibers. Scale bar: 5 µm.

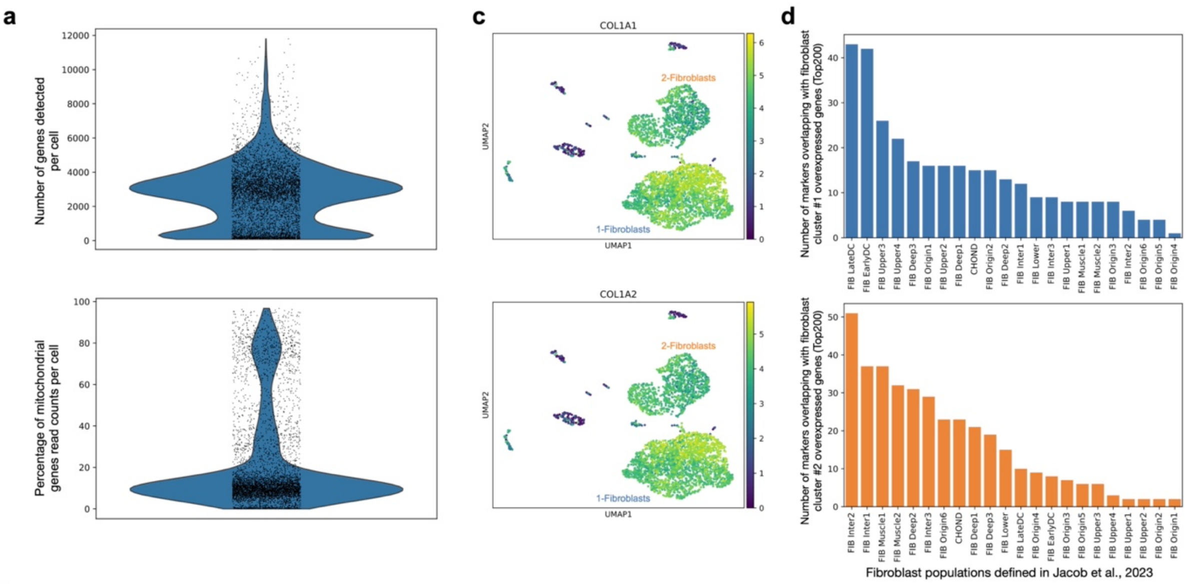

b

Sample Preparation	
Sample	Corn Snake Embryo sampled 25 days after Ovipositioning
Tissue	Dorsal skin
Single Cell Suspension Preparation	Williams & Sauka-Spengler, 2021
Library Preparation Kit	10X Chromium Single Cell 3' V3
**Sequencing**	
Total number of reads	235,545,093
Mean Reads per Cell	43,370
Median Genes per Cell	2,850
Median UMI Counts per Cell	10,357
Total Genes Detected	22,405
Valid Barcodes	97.80%
Valid UMIs	99.90%
Sequencing saturation	55.20%
Q30 Bases in RNA Read	87.10%
Q30 Bases in Barcode	95.90%
Q30 Bases in UMI	96.30%
**Mapping**	
Reads Mapped to Genome	92%
Reads Mapped Confidently to Genome	88.70%
Reads Mapped Confidently to Intergenic Regions	10.10%
Reads Mapped Confidently to Intronic Regions	13.80%
Reads Mapped Confidently to Exonic Regions	64.90%
Reads Mapped Confidently to Transcriptome	74.40%
Reads Mapped Antisense to Gene	4.20%
**Analysis Pipeline**	
Genome	UNIGE_PanGut_3.0; mtDNA accession: AM236349.1
Cell Ranger Version	7.0.0
Python Version	3.11.5
Scanpy Version	1.9.5
Cell Number	5,431
Include Features Detected in at Least this many Cells	3
Number of Genes per Cell Lower Threshold	1,000
Number of Genes per Cell Upper Threshold	9,000
Mitochondrial Genes per Cell Percentage Upper Threshold	20%
Predicted Doublets	36
Cell Number after Preprocessing	3,570
Nearest Neighbor Analysis (scanpy.pp.neighbors)	n_neighbors=10, n_pcs=16
Leiden Clustering Resolution	0.1

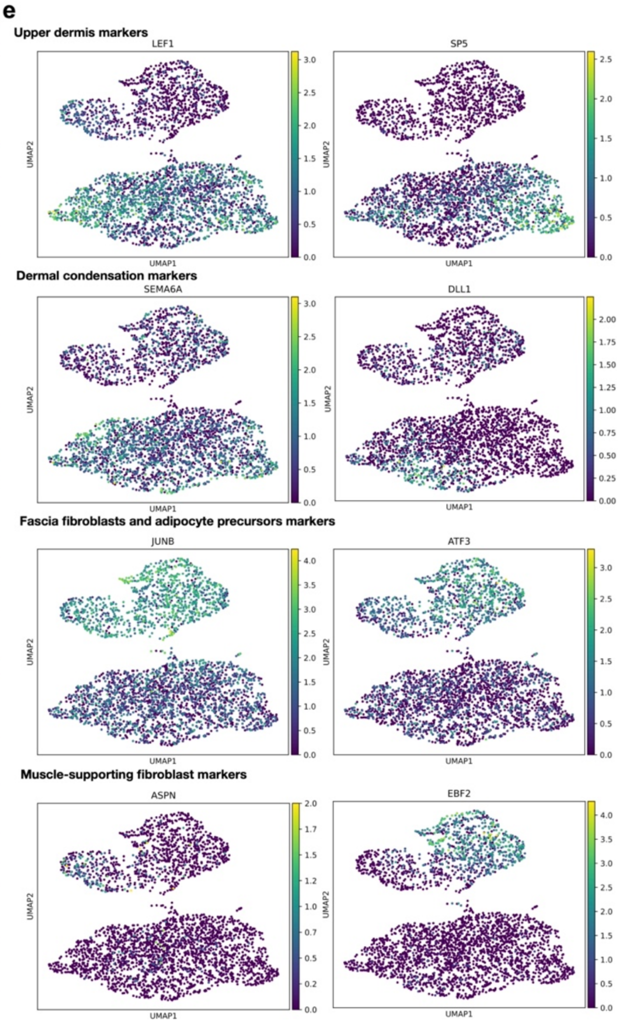

Supplementary Figure 9. Single-cell analyses metrics and cluster identification. (a) Violin plots depicting the distribution of the number of genes (top) and the percentage of mitochondrial UMIs (bottom) detected per cell. (b) Table listing key quality features of the single-cell RNA sequencing experiment, along with parameters and methods used for dataset processing. (c) UMAP embedding displaying the expression of collagen genes *COL1A1*

(*collagen type I alpha 1 chain*) and *COL1A2* (*collagen type I alpha 2 chain*). (d) Count of genes that intersect between the top 100 genes expressed in the two fibroblast clusters and the gene list specific to different mouse fibroblast types, as defined by Jacob et al., 2023. (e) UMAP embedding showing the expression of fibroblast markers in the two fibroblast clusters.

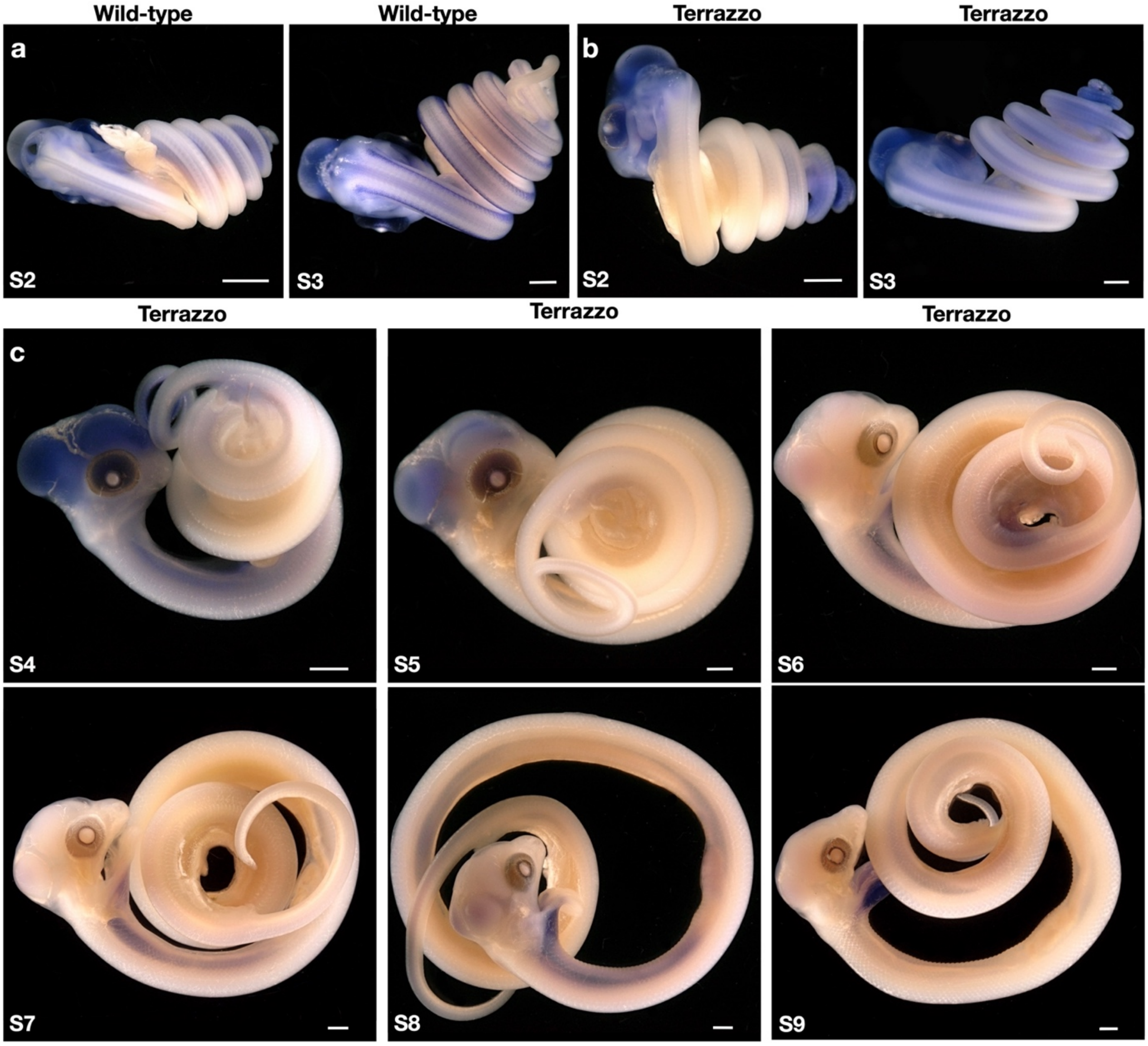

Supplementary Figure 10. Whole mount in situ hybridization of a *PMEL* probe on wild-type and Terrazzo individuals. Overall view of (a) wild-type embryos at S2 and S3, (b) Terrazzo embryos at S2 and S3, and (c) Terrazzo embryos at S4, S5, S6, S7, S8, and S9. No *PMEL* expression was detected in the Terrazzo embryos and the S2 wild-type embryo. At least two embryos were prepared for each stage. Scale bars: 1 mm.

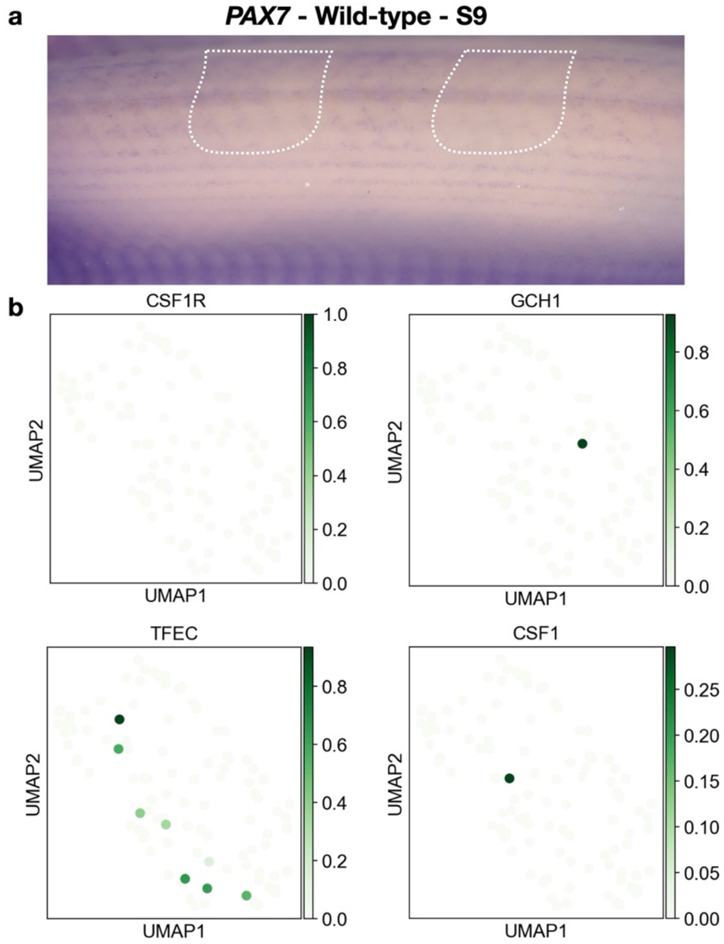

Supplementary Figure 11. Xanthophores and iridophores markers. (a) In situ hybridization of a *PAX7* probe, a classical marker of xanthophores in zebrafish, on a S9 wild-type embryo. With this method, we could detect very faint expression which seems to be in the background region, and not within the blotches. The regions that are likely to correspond to the blotches are delineated by a white dashed line. This result should be taken with caution. Species-specific xanthophores markers are needed for confirmation. (b) Expression levels of classical xanthophore (*CSF1R*, *GCH1*) and iridophore markers (*TFEC*, *CSF1*) are very low at this stage of development in corn snakes.

Supplementary Table 1. Genomic DNA libraries used for mapping the Terrazzo causative variant. In parentheses, we provide the total number of individuals in each library (column 'Source') and the average coverage for a 1.7 Gb genome (column 'Filtered reads'). PE: Paired-end reads.

**Source**	**Genotype**	**Library type**	**Total reads**	**Filtered reads**
Terrazzo male (1)	*tz/tz*	151 bp Illumina PE	185,920,113	179,764,993 (31.9x)
WT females (2)	*tz*/+	151 bp Illumina PE	173,071,991	167,429,684 (29.7x)
Terrazzo offspring (26)	*tz/tz*	151 bp Illumina PE	179,199,179	177,049,944 (31.4x)
WT offspring (19)	*tz*/+	151 bp Illumina PE	182,631,611	173,187,252 (30.8x)

Supplementary Table 2. List of the 35 protein coding genes in the Terrazzo interval on Super-scaffold 85. We provide information on the presence of co-segregating variants in the coding region of each gene ('SNPs/MNPs/indels' column) and of any non-synonymous amino-acid modifications in the protein coding part of the genes detected in the Terrazzo libraries when compared to the wild-type ('aa modifications' column). We include information on *APOH*, a differentially expressed gene in Terrazzo animals and located near, but not within, the reduced genomic interval.

Start	End	Transcript name	Gene name	SNPs/MNPs /indels	aa modifications
13037516	13048026	XM_034412574.1	*DNAJC14*	Yes	1
13062127	13076115	XM_034412579.1	*LOC117662879 - TMEM198-like*	No	No
13081057	13100003	XM_034413042.1	*MMP19*	Yes	2
13127686	13139693	XM_034412581.1	*PYM1*	No	No
13179802	13260083	XM_034412586.1	*DGKA*	Yes	No
**13267217**	**13287547**	**XM_034412587.1**	***PMEL***	**Yes**	**1 - Intron/Boundary**
13293248	13305583	XM_034412594.1	*CDK2*	Yes	No
13309147	13338581	XM_034412595.1	*RAB5B*	No	No
13339158	13355827	XM_034412591.1	*SUOX*	Yes	7
13365730	13446862	XM_034412597.1	*IKZF4*	Yes	No
13446148	13456495	XM_034412596.1	*LOC117662887 - ZNF397-like*	Yes	3
13460745	13464308	XM_034412601.1	*RPS26*	No	No
13476743	13580151	XM_034412602.1	*ERBB3*	Yes	No
13583701	13603199	XM_034412603.1	*PA2G4*	No	No
13609655	13615796	XM_034412607.1	*ZC3H10*	Yes	No
13619829	13699339	XM_034412608.1	*ERN1*	Yes	2
13712184	13772656	XM_034412610.1	*TEX2*	Yes	1
13846074	13846612	XM_034413059.1	*LOC117663130 - Nip7-like*	Yes	4
13875154	13918022	XM_034412617.1	*PECAM1*	Yes	No
13936200	13965732	XM_034413043.1	*MILR1*	Yes	1
13967547	13974282	XM_034412621.1	*POLG2*	Yes	2
13978131	13985240	XM_034412618.1	*DDX5*	No	No
13992707	14032286	XM_034412622.1	*CEP95*	Yes	5
14039938	14110709	XM_034412623.1	*SMURF2*	Yes	No
14134951	14142245	XM_034412627.1	*KPNA2*	Yes	No
14154592	14167596	XM_034412628.1	*CUNH17orf58*	Yes	3
14170574	14251567	XM_034412635.1	*BPTF*	Yes	6
14258194	14286601	XM_034412636.1	*NOL11*	Yes	No
14294181	14454961	XM_034412637.1	*PITPNC1*	Yes	No
14474205	14492143	XM_034412640.1	*PSMD12*	Yes	No
14504266	14642309	XM_034412646.1	*HELZ*	Yes	1
14654100	14681690	XM_034412648.1	*CACNG1*	No	No
14709064	14798889	XM_034412649.1	*CACNG4*	No	No
14892012	14922547	XM_034412650.1	*CACNG5*	Yes	No
14965065	15156109	XM_034412652.1	*PRKCA*	Yes	1
15230435	15248398	XM_034412653.1	*APOH*	No	No

Supplementary Table 3. ***PMEL*** **gene-editing results.** We provide information on the sequence modifications and phenotype of all the offspring. Highlighted in bold the unique heterozygous individual.

Sample	gRNA	Sequence modifications	Phenotype
EG.374.395.2023.1	204f	c.142_152del - p.S48PfsX68	Stripes
EG.374.395.2023.2	204f	c.151_157delins - p.[P51W;W53del]	WT
EG.374.395.2023.3	204f	None	WT
EG.374.395.2023.4	204f	c.[147T>C;149_151del] - p.Y50_P51delinsS	WT
EG.374.395.2023.5	204f	None	WT
EG.374.395.2023.6	204f	None	WT
EG.374.395.2023.7	204f	1056bp deletion + 639bp inversion	Stripes
EG.374.395.2023.8	204f	None	WT
EG.374.395.2023.9	204f	c.150_151insTCT - p.Y50_P51insS	WT
EG.374.395.2023.10	204f	None	WT
EG.377.232.2023.1	204f	c.143_155del - p.L49GfsX65	Stripes
EG.377.232.2023.2	204f	None	WT
EG.377.232.2023.3	204f	None	WT
EG.377.232.2023.4	204f	None	WT
EG.377.232.2023.5	204f	c.142_154del - p.S48GfsX65	Stripes
EG.377.232.2023.6	204f	c.145_157del - p.L49GfsX65	Stripes/Blotches
EG.377.232.2023.7	204f	c.152_154del - p.P50del	WT
EG.1179.210.2023.1	302r	c.238delA - p.I80X	Modified blotches
EG.1179.210.2023.2	302r	c.236_238del - p.T79del	WT
EG.1179.210.2023.3	302r	None	WT
EG.1179.210.2023.4	302r	c.237_261delinsA - p.I80_F87del	Stripes
EG.1179.210.2023.5	302r	c.238_239ins7 - p.I80SfsX98	Stripes
EG.1179.210.2023.6	302r	c.238_239insA - p.I80NfsX96	Stripes
EG.1179.210.2023.7	302r	None	WT
EG.1179.210.2023.8	302r	c.236_238del - p.T79del	WT
**EG.1179.210.2023.9**	**302r**	**c.[236_238del]+[203_249delins38] - p.[T79del]+[N68IfsX79]**	**WT**
EG.1179.210.2023.10	302r	c.234_239del - p.I80_T81del	WT
EG.1179.210.2023.11	302r	c.236_238del - p.T79del	WT
EG.1179.210.2023.12	302r	c.238_239del - p.I80NfsX95	Modified blotches

Supplementary Table 4. Primers and gRNAs used in this study.

Purpose	Orientation	Name	Sequence
PMEL transcript amplification	Forward	CSPMELF	ATGTCACGGATCTGGTTCCTATGGG
	Reverse	EG_PMELc_876R	TGGTCACCAAAATCCCAGGA
	Forward	EG_PMELc_701-F	TTCAGCATCACGGACCAGAT
	Reverse	EG_PMELc_1537-R	CACTGGGTTCATGGCTGTTG
	Forward	EG_PMELc_1321-F	GGGTGTGACTATTGCCATGTC
	Reverse	EG_PMELc_2090-R	GAGACATCATGGCCCGTAGA
	Forward	EG_PMELc_1887-F	GCCTCTACTGCGTCAACATC
	Reverse	EG_PMELc_2589-R	CATTTGTTAGAAGACCTGGCCA
	Forward	EG_PMELc_2370-F	GGGTGGGGAGATACAGACAG
	Reverse	EG_PMELc_3039-R	ACAGAGATGCTCCATTGGCT
Genotyping	Forward	EG_sc85-2900655-F	TTTCATAGCGGTGATGGCCT
	Reverse	EG_sc85-2901018-R	TGTCCTCCCACTGAAAGACA
	Forward	EG_sc85-14891026-F	TGCTCAGATTAGAGTGCGCA
	Reverse	EG_sc85-14891444-R	TGGGACATTCAAACTTCCCTT
PMEL qPCR (exons 3-4)	Forward	EG_PMEL_qF3_qPCR	TCAGGATTGCTTTGCGCTTT
	Reverse	EG_PMEL_qF3_qPCR	ACGGGTGCCATTTATGATGC
PMEL qPCR (exons 8-10)	Forward	EG_PMEL_qF2_qPCR	CTGGACATCATCCAGGGCAT
	Reverse	EG_PMEL_qF2_qPCR	CCCAAGAAGTCTGCACAACC
PMEL in situ probe	Forward	EG_PMELc_134F	AACAACAGATGGGGGCAGAA
	Reverse	EG_PMELc_867R	TTTGGTGACCAGAGTGGGAC
MLANA in situ probe	Forward	EG_MLANAc-49-F	GCTTGACTTGATTGGCAGCT
	Reverse	EG_MLANAc-524-R	AGCTCGTGTTTTCTCTCCCA
GPNMB in situ probe	Forward	EG_GPNMBc-1134-F	TTGACCAGTGATAGCCCAGC
	Reverse	EG_GPNMBc-1761-R	ACCCACTGCCATCTCCAAAA
PAX7 in situ probe	Forward	EG_PAX7_15F	CAGCATCGATGGCATCTTGG
	Reverse	EG_PAX7_475R	TGAAGCTGTCCGAGTAGCTG
CRISPR-Cas9 gene-editing	gRNA	EG_PMEL_gRNA_204f	GGAACTCCAGTCTTTACCCCCGG
	Forward	EG_PMELtg_17522-F	GGTCACTCTCACCACCAACA
	Reverse	EG_PMELtg_17938-R	ACTCCAGCTTTGCCTGTTCT
	gRNA	EG_PMEL_gRNA_302r	ACTTTGGCACTTGTTATAGTCGG
	Forward	EG_PMELtg_16548-F	AGTCCCCTAAGTGTCCAAGC
	Reverse	EG_PMELtg_17072-R	CGTGCCCTGTTTTATCCTCG
