# Supplementary material for: PMEL is involved in snake colour pattern transition from blotches to stripes
Source: Nat Commun. 2024 Sep 3;15:7655. doi: 10.1038/s41467-024-51927-0 (PMC11371805; doi:10.1038/s41467-024-51927-0)
Supplement: Supplementary file 3 — Description of Additional Supplementary Files [file 41467_2024_51927_MOESM3_ESM.pdf]

Description of Additional Supplementary Files

File Name: Supplementary Data 1
Description: Significantly upregulated genes in each cluster. Significantly differentially expressed genes (adjusted p-value ≤ 0.05) identified with two-sided scanpy Wilcoxon rank-sum test (sc.tl.rank_genes_groups). Correction was performed using the Benjamini and Hochberg procedure.
