# Supplementary material for: PMEL is involved in snake colour pattern transition from blotches to stripes
Source: Nat Commun. 2024 Sep 3;15:7655. doi: 10.1038/s41467-024-51927-0 (PMC11371805; doi:10.1038/s41467-024-51927-0)
Supplement: Supplementary file 5 — Reporting Summary [file 41467_2024_51927_MOESM5_ESM.pdf]

nature portfolio

Corresponding author(s): Athanasia TZIKA
Last updated by author(s): Jun 24, 2024

Reporting Summary

Nature Portfolio wishes to improve the reproducibility of the work that we publish. This form provides structure for consistency and transparency in reporting. For further information on Nature Portfolio policies, see our Editorial Policies and the Editorial Policy Checklist.

Statistics

For all statistical analyses, confirm that the following items are present in the figure legend, table legend, main text, or Methods section.

n/a	Confirmed	
☐	☒	The exact sample size (*n*) for each experimental group/condition, given as a discrete number and unit of measurement
☐	☒	A statement on whether measurements were taken from distinct samples or whether the same sample was measured repeatedly
☐	☒	The statistical test(s) used AND whether they are one- or two-sided<br>*Only common tests should be described solely by name; describe more complex techniques in the Methods section.*
☒	☐	A description of all covariates tested
☐	☒	A description of any assumptions or corrections, such as tests of normality and adjustment for multiple comparisons
☐	☒	A full description of the statistical parameters including central tendency (e.g. means) or other basic estimates (e.g. regression coefficient) AND variation (e.g. standard deviation) or associated estimates of uncertainty (e.g. confidence intervals)
☐	☒	For null hypothesis testing, the test statistic (e.g. *F*, *t*, *r*) with confidence intervals, effect sizes, degrees of freedom and *P* value noted<br>*Give P values as exact values whenever suitable.*
☒	☐	For Bayesian analysis, information on the choice of priors and Markov chain Monte Carlo settings
☒	☐	For hierarchical and complex designs, identification of the appropriate level for tests and full reporting of outcomes
☒	☐	Estimates of effect sizes (e.g. Cohen's *d*, Pearson's *r*), indicating how they were calculated

Our web collection on statistics for biologists contains articles on many of the points above.

Software and code

Policy information about availability of computer code

Data collection	N/A
Data analysis	FASTQC, sickle v1.33, SAMtools v1.9, Platypus v0.8.1, STAR (v2.7.0d), R packages: Rsubread (v2.2.1), edgeR package (v3.40.2), DESeq2 package (v1.38.3), Cellranger (v7.0.0), Scrublet v0.2.3, scanpy, IMOD software

For manuscripts utilizing custom algorithms or software that are central to the research but not yet described in published literature, software must be made available to editors and reviewers. We strongly encourage code deposition in a community repository (e.g. GitHub). See the Nature Portfolio guidelines for submitting code & software for further information.

Data

Policy information about availability of data

All manuscripts must include a data availability statement. This statement should provide the following information, where applicable:

- Accession codes, unique identifiers, or web links for publicly available datasets
- A description of any restrictions on data availability
- For clinical datasets or third party data, please ensure that the statement adheres to our policy

The sequencing data generated in this study have been deposited in NCBI under accession code PRJNA1073516 [https://www.ncbi.nlm.nih.gov/bioproject/PRJNA1073516/] for the genomic sequencing, GSE262160 [https://www.ncbi.nlm.nih.gov/geo/query/acc.cgi?acc=GSE262160] for the single-cell and GSE262159 [https://www.ncbi.nlm.nih.gov/geo/query/acc.cgi?acc=GSE262159] for the bulk RNA sequencing data.

Research involving human participants, their data, or biological material

Policy information about studies with human participants or human data. See also policy information about sex, gender (identity/presentation), and sexual orientation and race, ethnicity and racism.

Reporting on sex and gender	N/A
Reporting on race, ethnicity, or other socially relevant groupings	N/A
Population characteristics	N/A
Recruitment	N/A
Ethics oversight	N/A

Note that full information on the approval of the study protocol must also be provided in the manuscript.

Field-specific reporting

Please select the one below that is the best fit for your research. If you are not sure, read the appropriate sections before making your selection.

☒ Life sciences ☐ Behavioural & social sciences ☐ Ecological, evolutionary & environmental sciences

For a reference copy of the document with all sections, see nature.com/documents/nr-reporting-summary-flat.pdf

Life sciences study design

All studies must disclose on these points even when the disclosure is negative.

Sample size	Mapping-by-sequencing: based on our past experience, a minimum of 10 homozygous and 10 heterozygous offspring are needed, taking into account that corn snakes are seasonal breeders laying eggs once per year. Here we use 26 homozygous and 19 heterozygous.<br>transcriptomics: only embryos of the same clutch were sampled to ensure a uniform genetic background. Clutches range from 15 to 20 eggs. A minimum of three samples per genotype were run.<br>Gene-editing: the follicles of two females were injected to make sure that the outcome is independent of the genetic background of each female.<br>TEM: three blocks were prepared for each type of sample.<br>WISH: at least two embryos per stage<br>No sample size calculations were performed.
Data exclusions	No data was excluded from the analyses.
Replication	Mapping-by-sequencing: we confirmed the results by genotyping additional individuals<br>transcriptomics: embryos from different clutches were analysed<br>All attempts at replication were successful.
Randomization	Randomization is not relevant to this study that aims to compare wild-type to mutants.
Blinding	Blinding is not relevant to this study that aims to compare wild-type to mutants.

Reporting for specific materials, systems and methods

We require information from authors about some types of materials, experimental systems and methods used in many studies. Here, indicate whether each material, system or method listed is relevant to your study. If you are not sure if a list item applies to your research, read the appropriate section before selecting a response.

Materials & experimental systems

n/a	Involved in the study
☒	☐ Antibodies
☒	☐ Eukaryotic cell lines
☒	☐ Palaeontology and archaeology
☐	☒ Animals and other organisms
☒	☐ Clinical data
☒	☐ Dual use research of concern
☒	☐ Plants

Methods

n/a	Involved in the study
☒	☐ ChIP-seq
☒	☐ Flow cytometry
☒	☐ MRI-based neuroimaging

Animals and other research organisms

Policy information about studies involving animals; ARRIVE guidelines recommended for reporting animal research, and Sex and Gender in Research

Laboratory animals	Embryos at different developmental stages (from E20-E30), juveniles and adult corn snakes Pantherophis guttatus were used.
Wild animals	N/A
Reporting on sex	We report the sex of the embryos used for bulk RNAseq. For the remaining of the analyses, the sex is not relevant.
Field-collected samples	N/A
Ethics oversight	Sampling and imaging were performed under the experimentation permits GE24/33145 and GE150/34215 provided by cantonal veterinary authorities of Geneva, Switzerland.

Note that full information on the approval of the study protocol must also be provided in the manuscript.

Plants

Seed stocks	N/A
Novel plant genotypes	N/A
Authentication	N/A
